# Supplementary material for: Evidence for wastewaters as environments where mobile antibiotic resistance genes emerge
Source: Commun Biol. 2023 Mar 25;6:321. doi: 10.1038/s42003-023-04676-7 (PMC10039890; doi:10.1038/s42003-023-04676-7)
Supplement: Supplementary file 2 — Supplementary Material [file 42003_2023_4676_MOESM2_ESM.pdf]

Supplementary information

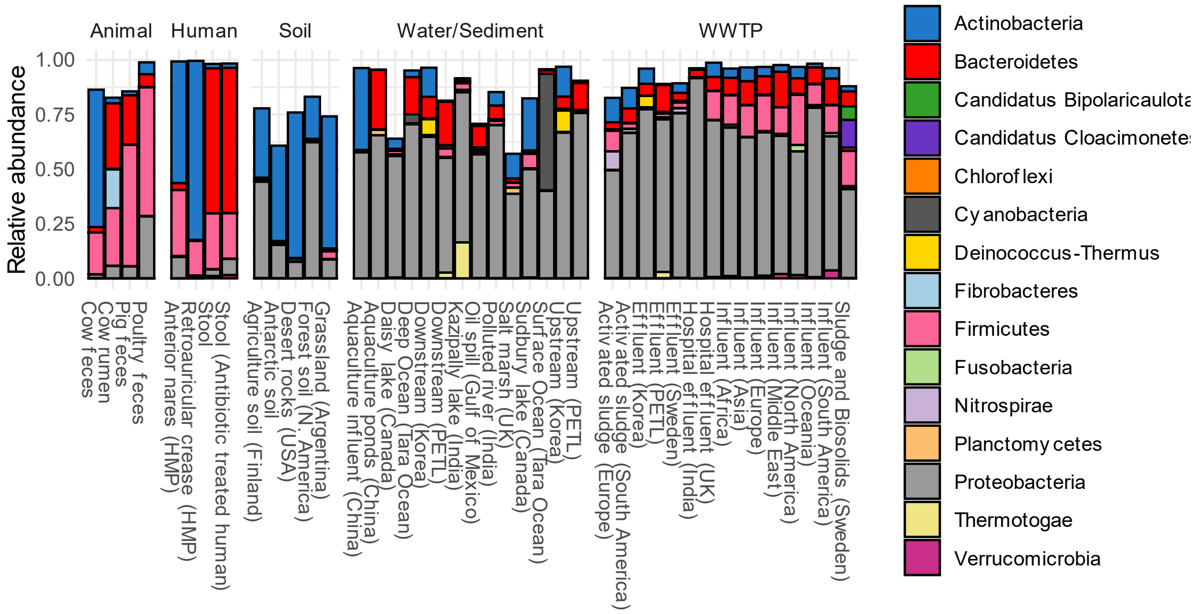

Supplementary figure 1: The phyla composition of the investigated environments.

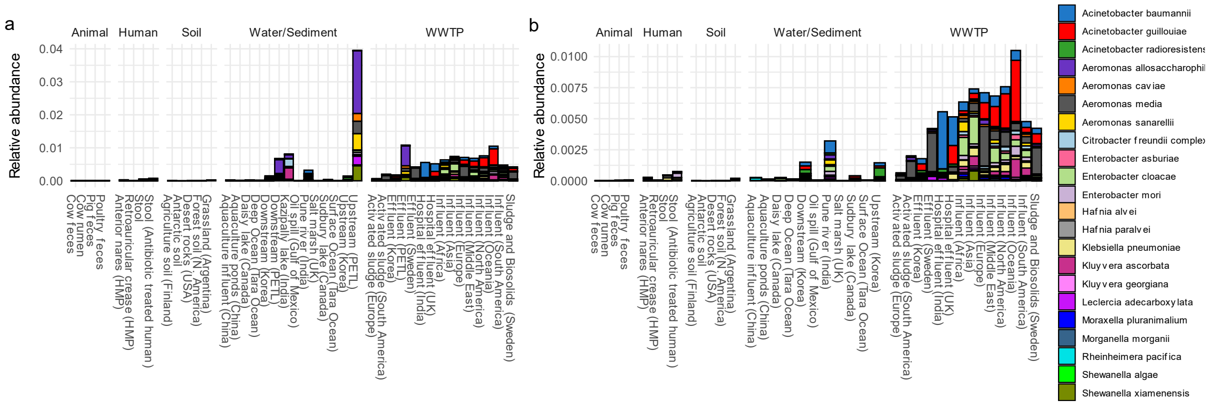

Supplementary figure 2: Barplot of the average relative abundance of known origin species for mobile antibiotic resistance genes in all environments (a) and with PETL and Kazipally lake excluded (b).

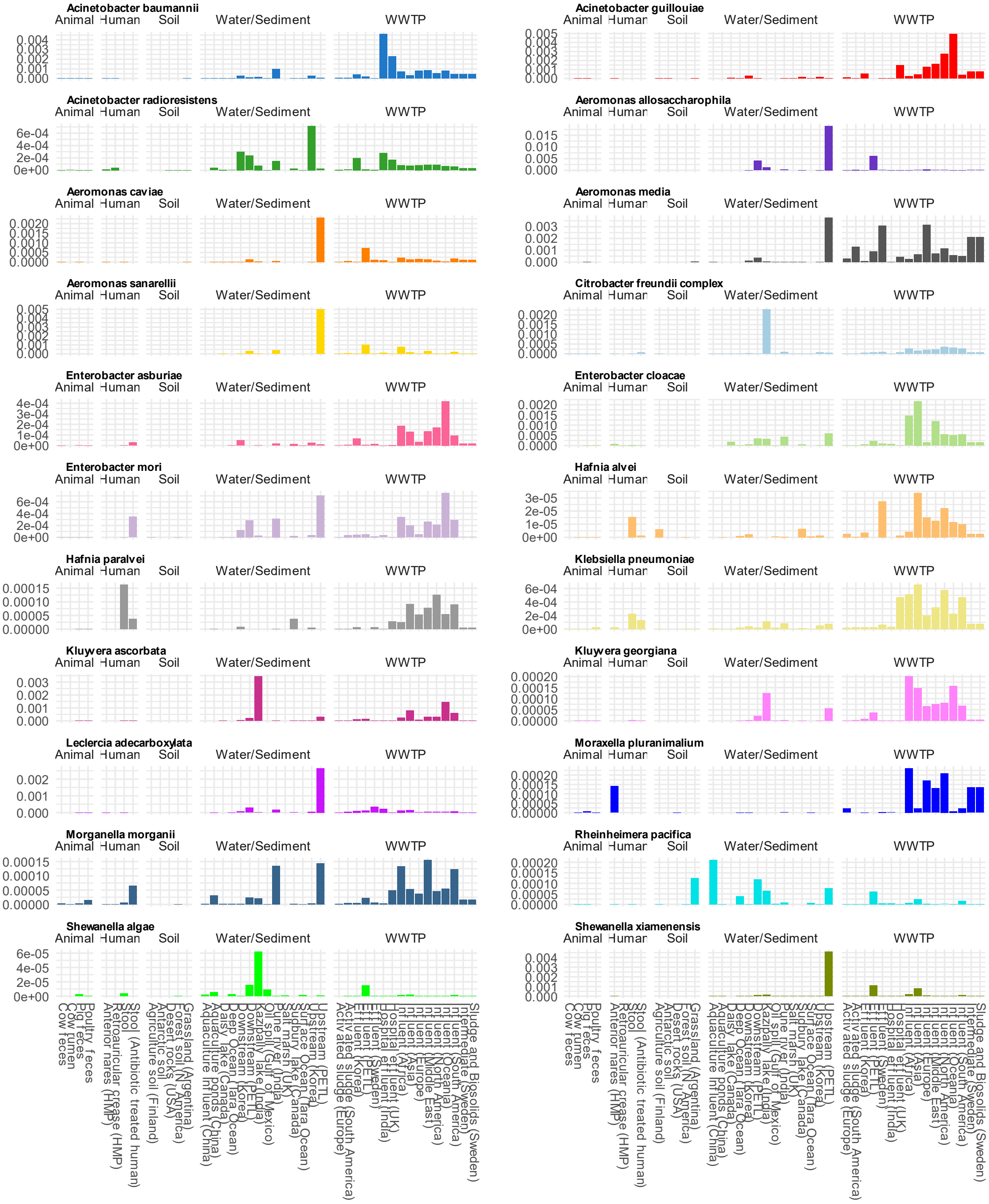

Supplementary figure 3: Average relative abundance of known origin species for mobile antibiotic resistance genes in the different environments.

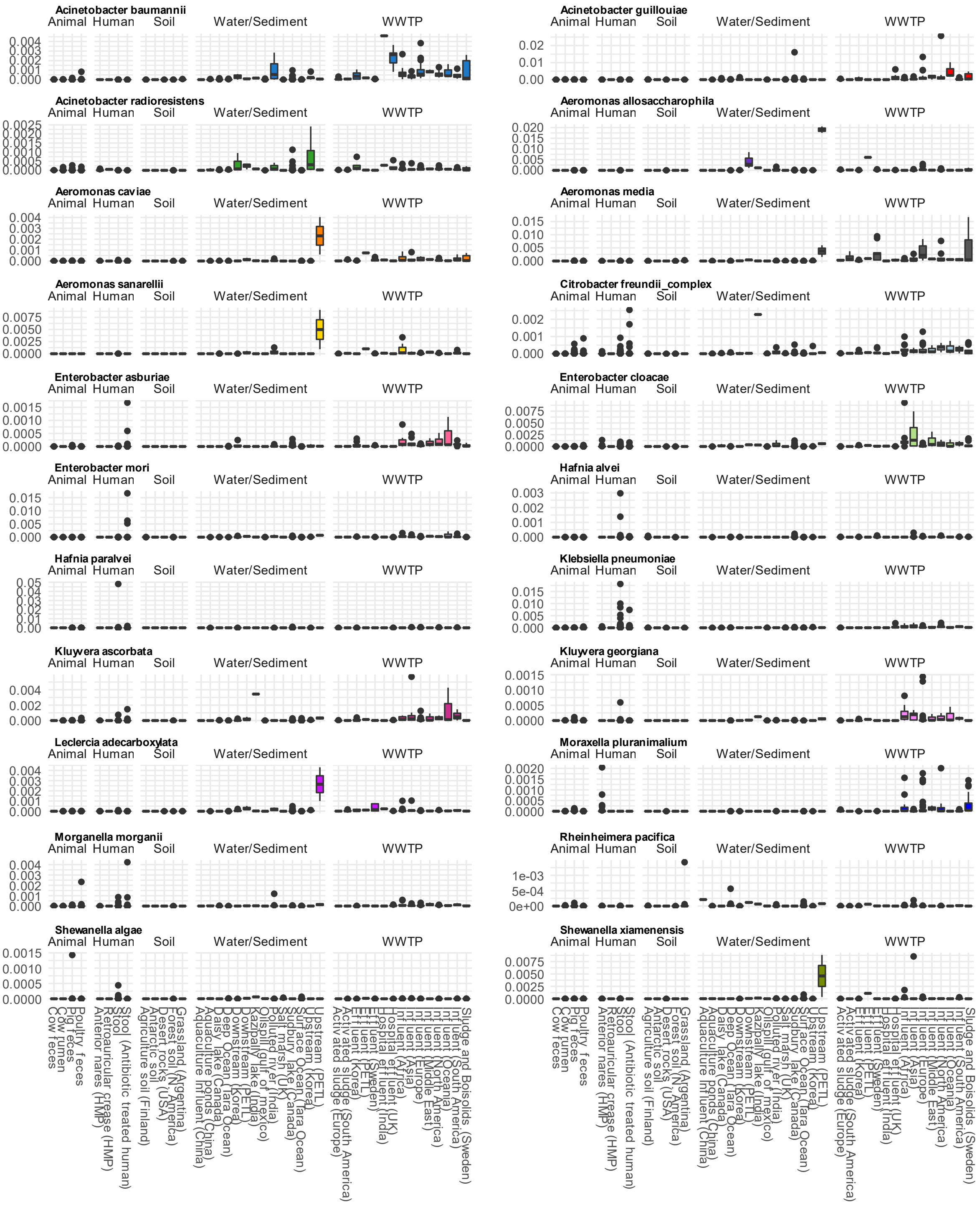

Supplementary Figure 4: Relative abundance of known origin species for mobile antibiotic resistance genes in all samples.

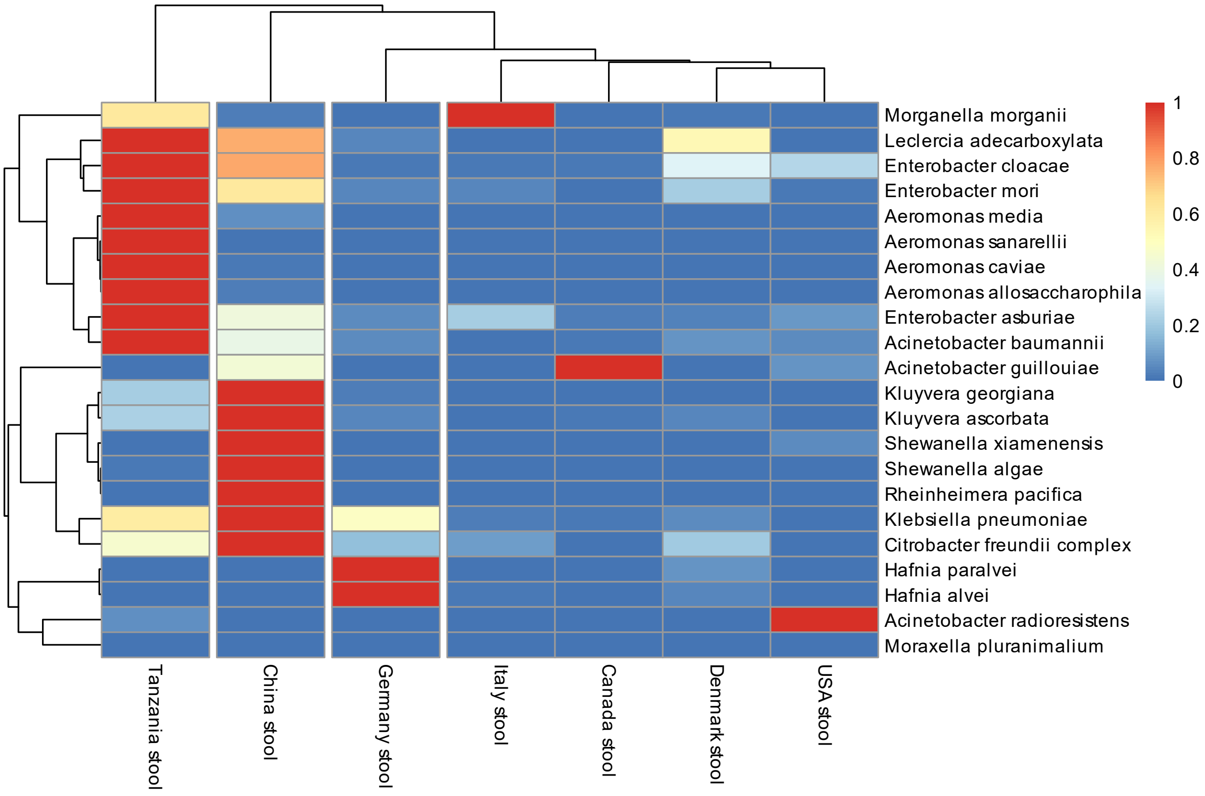

Supplementary figure 5: Abundance of known origin species of mobile antibiotic resistance genes in stool samples. The presented data was normalized by the maximum relative abundance for each species. Samples from participants marked as being treated with antibiotics were excluded.

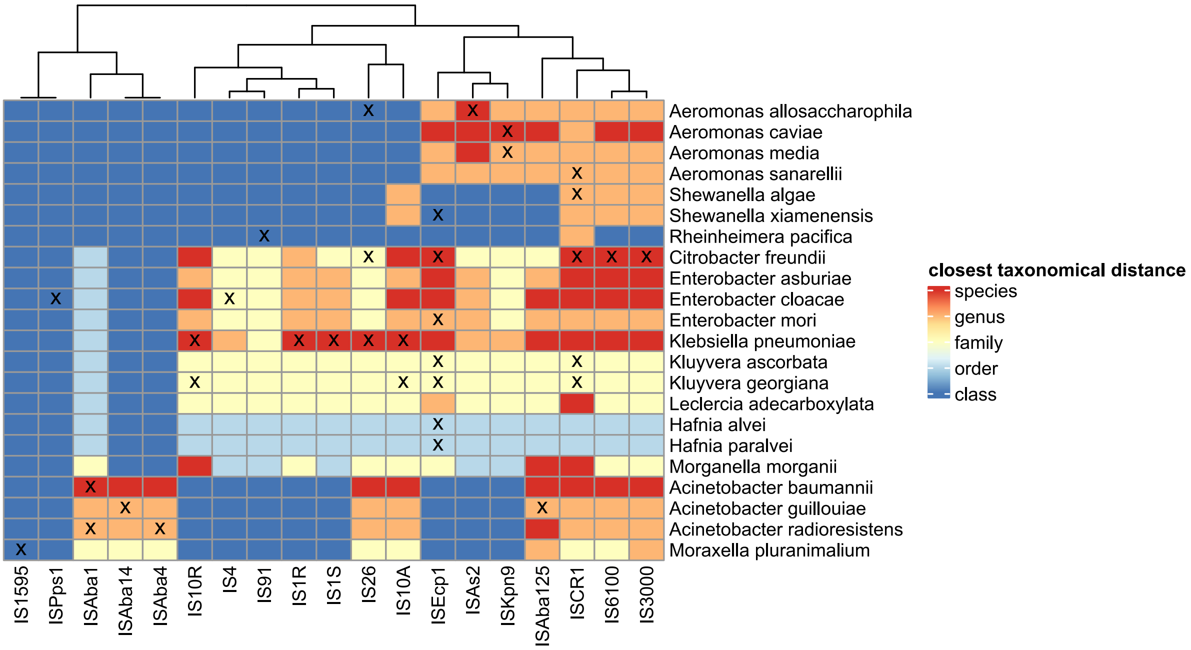

Supplementary figure 6: Closest taxonomical distance between species carrying MISE and origin species. A cross indicated that a MISE has been associated with mobilization of an ARG from the corresponding species.

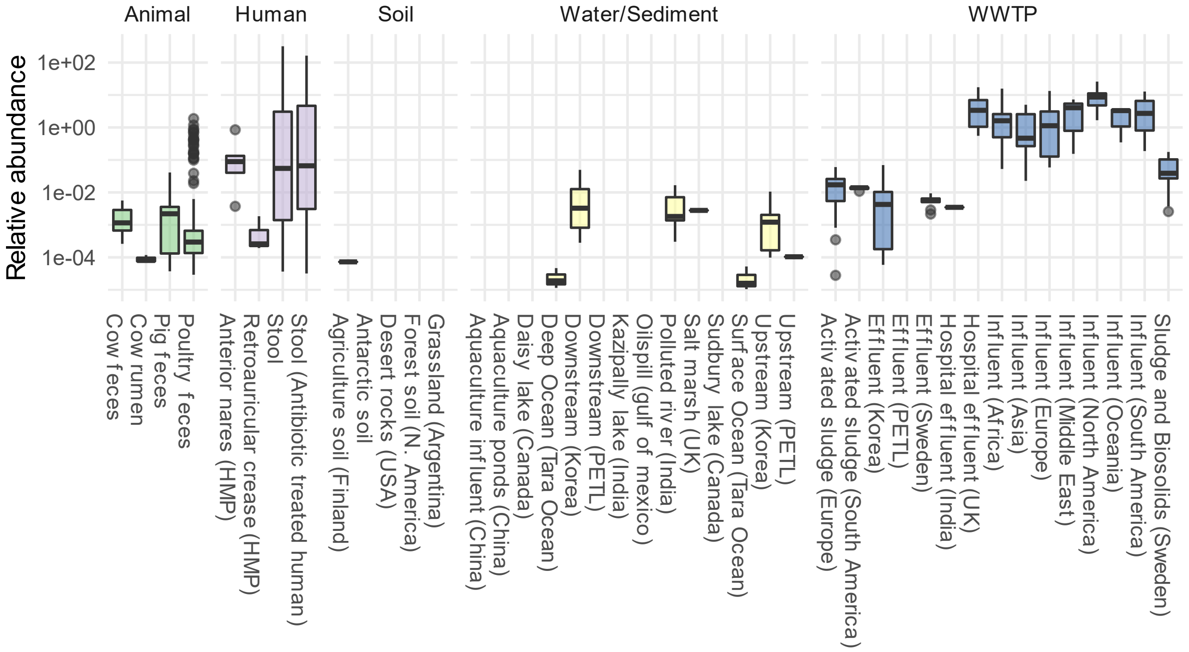

Supplementary figure 7: Relative abundance of crAssphage in all investigated samples.

Supplementary table 1: Fraction identified fragments from genomes of known origin species for mobile antibiotic resistance genes and fraction of fragments that were classified at a species taxonomical level.

			TPR				Fraction identified species			
			Genome in DB		Genome not in DB		Genome in DB		Genome not in DB	
	# genomes in DB	# Genomes not in DB	100bp	150bp	100bp	150bp	100bp	150bp	100bp	150bp
Acinetobacter baumannii	20	9	0.99	0.99	1.00	1.00	0.09	0.07	0.10	0.08
Acinetobacter guillouiae	1	7	1.00	1.00	0.57	0.57	0.97	0.97	0.61	0.63
Acinetobacter radioresistens	4	0	0.75	0.75	1.00	1.00	0.96	0.96	0.88	0.89
Aeromonas allosaccharophila	1	9	1.00	1.00	0.97	0.98	0.55	0.54	0.47	0.46
Aeromonas caviae	4	11	1.00	1.00	0.80	0.76	0.11	0.09	0.05	0.04
Aeromonas media	7	2	0.85	0.85	0.98	0.98	0.56	0.56	0.49	0.51
Aeromonas sanarelli	1	0	1.00	1.00	0.94	0.95	0.72	0.73	0.34	0.35
Citrobacter freundii	17	23	1.00	1.00	0.89	0.87	0.06	0.05	0.05	0.04
Enterobacter asburiae	7	6	1.00	1.00	0.89	0.88	0.14	0.13	0.08	0.06
Enterobacter cloacae	12	3	1.00	1.00	0.57	0.57	0.36	0.36	0.21	0.21
Enterobacter mori	1	8	1.00	1.00	1.00	1.00	0.72	0.73	0.57	0.58
Hafnia alvei	6	15	0.79	0.79	0.88	0.88	0.33	0.32	0.27	0.26
Hafnia paralvei	1	18	1.00	1.00	0.98	0.98	0.97	0.97	0.84	0.85
Klebsiella pneumoniae	19	14	1.00	1.00	0.99	0.99	0.05	0.04	0.05	0.04
Kluyvera ascorbata	1	3	1.00	1.00	1.00	1.00	0.87	0.88	0.86	0.87
Kluyvera georgiana	1	1	1.00	1.00	1.00	1.00	0.68	0.70	0.73	0.75
Leclercia adecarboxylata	10	1	1.00	1.00	0.99	0.99	0.27	0.21	0.25	0.18
Moraxella pluranimalium	1	0	1.00	1.00	-	-	0.99	0.99	-	-
Morganella morganii	13	3	1.00	1.00	1.00	1.00	0.97	0.97	0.97	0.97
Rheinheimera pacifica	1	0	1.00	1.00	-	-	0.99	0.99	-	-
Shewanella algae	4	2	1.00	1.00	0.99	0.99	0.66	0.66	0.52	0.51
Shewanella xianamenensis	1	13	1.00	1.00	0.99	0.99	0.33	0.31	0.33	0.31
**Average**			**0.97**	**0.97**	**0.92**	**0.92**	**0.56**	**0.56**	**0.43**	**0.43**

Supplementary table 2: False positive rates (FPR) estimated from CAMISIM high and medium complexity simulated metagenomes. The FPR was calculated in two ways: divided by total number of fragments identified at the bacterial species level (first two columns) and divided by total number of identified bacterial fragments.

	Ref: classified species		Ref: identified bacterial fragments	
#Species	FPR	Fraction identified species (any)	FPR	Fraction identified bacteria (any)
Acinetobacter baumannii	1.30E-05	0.35	7.71E-06	0.61
Acinetobacter guillouiae	2.69E-06	0.35	1.56E-06	0.61
Acinetobacter gyllenbergii	0	0.35	0	0.61
Acinetobacter radioresistens	7.81E-08	0.35	4.56E-08	0.61
Aeromonas allosaccharophila	0	0.35	0	0.61
Aeromonas caviae	0	0.35	0	0.61
Aeromonas media	0	0.35	0	0.61
Aeromonas sanarelli	0	0.35	0	0.61
Citrobacter freundii	4.33E-06	0.35	2.53E-06	0.61
Enterobacter asburiae	3.56E-09	0.35	2.07E-09	0.61
Enterobacter cloacae	1.57E-07	0.35	8.68E-08	0.61
Enterobacter mori	1.42E-08	0.35	8.26E-09	0.61
Hafnia alvei	0	0.35	0	0.61
Hafnia paralvei	0	0.35	0	0.61
Klebsiella pneumoniae	1.03E-07	0.35	6.04E-08	0.61
Kluyvera ascorbata	0	0.35	0	0.61
Kluyvera georgiana	0	0.35	0	0.61
Leclercia adecarboxylata	0	0.35	0	0.61
Moraxella pluranimalium	0	0.35	0	0.61
Morganella morganii	1.67E-06	0.35	9.76E-07	0.61
Rheinheimera pacifica	0	0.35	0	0.61
Shewanella algae	0	0.35	0	0.61
Shewanella xianamenensis	0	0.35	0	0.61
**Average**	**9.59E-07**	**0.35**	**5.64E-07**	**0.61**

Supplementary table 3: False positive rate (FPR) of genomes of the same genera as the corresponding origin species, where none of the tested genomes were included in the database. FPR was calculated as total number of fragments falsely classified as an origin species divided by the total number fragments that were classified at species level.

	# Genomes	FPR		Fraction classified bacterial species	
		100bp	150bp	100bp	150bp
Acinetobacter baumannii	14	1.31E-02	1.26E-02	0.40	0.39
Acinetobacter guillouiae	23	3.96E-05	1.03E-05	0.28	0.27
Acinetobacter radioresistens	23	8.00E-04	7.19E-04	0.28	0.27
Aeromonas allosaccharophila	6	4.90E-03	4.70E-03	0.14	0.13
Aeromonas caviae	3	1.34E-03	6.60E-04	0.33	0.31
Aeromonas media	6	4.69E-02	5.71E-02	0.10	0.08
Aeromonas sanarelli	7	0.00E+00	0.00E+00	0.16	0.15
Citrobacter freundii	6	2.76E-02	3.47E-02	0.20	0.19
Enterobacter asburiae	13	3.30E-03	3.50E-03	0.17	0.17
Enterobacter cloacae	10	5.27E-02	5.74E-02	0.09	0.09
Enterobacter mori	13	1.50E-03	1.10E-03	0.17	0.17
Hafnia alvei	2	9.00E-03	1.95E-02	0.44	0.44
Hafnia paralvei	3	1.49E-02	1.31E-03	0.18	0.17
Klebsiella pneumoniae	12	6.81E-02	7.05E-02	0.38	0.38
Kluyvera ascorbata	0	-	-	-	-
Kluyvera georgiana	1	0.00E+00	0.00E+00	0.88	0.89
Leclercia adecarboxylata	0	-	-	-	-
Moraxella pluranimalium	2	0.00E+00	0.00E+00	0.87	0.88
Morganella morganii	0	-	-	-	-
Rheinheimera pacifica	0	-	-	-	-
Shewanella algae	9	1.50E-03	2.78E-03	0.14	0.14
Shewanella xianamenensis	10	0.00E+00	0.00E+00	0.18	0.17
Average		1.36E-02	1.48E-02	0.30	0.29

Supplementary table 4: European Nucleotide Archive project accession numbers to the datasets used in this study.

Dataset source/ name	Type	ENA project accession number	Secondary accession
**Human**			
HMP	Various body sites	PRJNA48479	SRP002163
PD gut	Stool	PRJEB17784	ERP019674
Antibiotic treated human	Stool	PRJEB20800	ERP022986
Ceph. treated human	Stool	PRJEB8094	ERP009131
Hadza hunter	Stool	PRJNA278393	SRP056480
DB gut	Stool	PRJNA422434	SRP008047
Italian youth	Stool	PRJNA278393	SRP056480
**Water/Sediment**			
Aquaculture	Aquaculture (water from various sites)	PRJEB22134	ERP024470
Pune river	Polluted river sediments	-	MGRAST19878
Oil spill	Ocean deep water	PRJEB14900	ERP016581
Tara Ocean	Ocean deep + surface water	PRJEB1787	ERP001736
Indian lake	Polluted lake sediment	PRJEB6102	ERP005569
Daisy lake	Lake sediment	PRJEB14421	ERP016063
Sudbury lake	Lake sediment	PRJEB18063	ERP019980
Salt marsh	Salt marsh sediment	PRJEB19235	ERP021219
**WWTP**			
Global sewage	Influent	PRJEB13831	ERP015409
Swedish WWTP	Various sites	PRJEB14051	ERP015657
Korea WWTP	Various sites	PRJNA506137	SRP170004
Hospital effluent UK	Hospital effluent	PRJEB34410	ERP117308
Hospital effluent India	Hospital effluent	PRJNA497765	SRP166249
Activated sludge 1	Activated sludge	PRJEB8087	ERP009124
Activated sludge 2	Activated sludge	PRJNA288131	SRP060024
**Soil**			
Agriculture soil	Agriculture soil	PRJEB22376	ERP104056
Forest soil	Forest soil	PRJEB8420	ERP009498
Desert	Desert rocks	PRJEB13142	ERP014680
Grassland	Partially polluted soil	PRJEB22053	ERP024378
**Animal**			
Pig gut	Feces pig	PRJEB11755	ERP013165
Pig and poultry	Feces pig and poultry	PRJEB22062	ERP024389
Cow feces	Feces	PRJNA485488	SRP157238
Cow rumen	Rumen	PRJEB23561	ERP105320

Supplementary table 5: The environments in which the average relative abundance was the highest for each origin species, with the pharmaceutical polluted environments PETL excluded.

Species	Environment	Average relative abundance
Acinetobacter radioresistens	Upstream (Korea)	0.000717
Acinetobacter guillouiae	Influent (Oceania)	0.004931
Shewanella xiamenensis	Influent (Asia)	0.00079
Acinetobacter baumannii	Hospital effluent (India)	0.004598
Enterobacter cloacae	Influent (Asia)	0.002175
Aeromonas allosaccharophila	Influent (Europe)	0.000367
Moraxella pluranimalium	Influent (Africa)	0.000237
Klebsiella pneumoniae	Influent (Asia)	0.000655
Leclercia adecarboxylata	Effluent (Sweden)	0.000339
Aeromonas media	Influent (Europe)	0.003173
Rheinheimera pacifica	Aquaculture influent (China)	0.000211
Aeromonas caviae	Influent (Africa)	0.00023
Hafnia alvei	Influent (Asia)	3.37E-05
Enterobacter mori	Influent (Oceania)	0.000749
Morganella morganii	Influent (Middle East)	0.000156
Shewanella algae	Oilspill (gulf of mexico)	9.03E-06
Kluyvera ascorbata	Influent (Oceania)	0.001469
Kluyvera georgiana	Influent (Africa)	0.0002
Enterobacter asburiae	Influent (Oceania)	0.000419
Hafnia paralvei	Stool	0.000163
Aeromonas sanarellii	Influent (Africa)	0.0008
Citrobacter freundii_complex	Influent (North America)	0.000348

Supplementary table 6: Results from Fisher's exact test on presence data for human stool and wastewater treatment plant influent.

species	FDR	OR
Acinetobacter_baumannii	6.55E-114	Inf
Acinetobacter_guillouiae	3.67E-109	Inf
Aeromonas_media	3.88E-107	Inf
Acinetobacter_radioresistens	9.98E-96	5291.169
Aeromonas_caviae	8.69E-91	3850.987
Aeromonas_allosaccharophila	4.25E-88	Inf
Citrobacter_freundii_complex	7.31E-69	182.1987
Enterobacter_asburiae	5.97E-64	168.8516
Enterobacter_cloacae	3.96E-61	100.1642
Kluyvera_ascorbata	1.47E-60	116.5027
Morganella_morganii	4.32E-60	155.3092
Shewanella_xiamenensis	2.57E-56	Inf
Enterobacter_mori	2.82E-56	106.7644
Klebsiella_pneumoniae	4.32E-51	101.9082
Aeromonas_sanarellii	3.39E-48	565.5392
Leclercia_adecarboxylata	9.13E-46	275.4859
Hafnia_paralvei	1.60E-39	50.92588
Kluyvera_georgiana	9.08E-35	123.7277
Moraxella_pluranimalium	3.52E-33	Inf
Hafnia_alvei	3.81E-13	19.41957
Rheinheimera_pacifica	2.77E-05	Inf
Shewanella_algae	0.361772	0
