# Supplementary material for: Evidence for wastewaters as environments where mobile antibiotic resistance genes emerge
Source: Commun Biol. 2023 Mar 25;6:321. doi: 10.1038/s42003-023-04676-7 (PMC10039890; doi:10.1038/s42003-023-04676-7)
Supplement: Supplementary file 3 — Description of Additional Supplementary Files [file 42003_2023_4676_MOESM3_ESM.pdf]

# Description of Additional Supplementary Files

**File name:** Supplementary Data 1

**Description:** Accession numbers to assemblies used in this study.

**File name:** Supplementary Data 2

**Description:** The source data behind the figures in the paper.
