# Supplementary material for: Evidence for wastewaters as environments where mobile antibiotic resistance genes emerge
Source: Commun Biol. 2023 Mar 25;6:321. doi: 10.1038/s42003-023-04676-7 (PMC10039890; doi:10.1038/s42003-023-04676-7)
Supplement: Supplementary file 6 — Reporting Summary [file 42003_2023_4676_MOESM6_ESM.pdf]

nature portfolio

Corresponding author(s): Joakim Larsson and Fanny Berglund

Last updated by author(s): Feb 22, 2023

Reporting Summary

Nature Portfolio wishes to improve the reproducibility of the work that we publish. This form provides structure for consistency and transparency in reporting. For further information on Nature Portfolio policies, see our Editorial Policies and the Editorial Policy Checklist.

Statistics

For all statistical analyses, confirm that the following items are present in the figure legend, table legend, main text, or Methods section.

n/a	Confirmed	
☐	☒	The exact sample size (*n*) for each experimental group/condition, given as a discrete number and unit of measurement
☒	☐	A statement on whether measurements were taken from distinct samples or whether the same sample was measured repeatedly
☐	☒	The statistical test(s) used AND whether they are one- or two-sided<br>*Only common tests should be described solely by name; describe more complex techniques in the Methods section.*
☐	☒	A description of all covariates tested
☐	☒	A description of any assumptions or corrections, such as tests of normality and adjustment for multiple comparisons
☒	☐	A full description of the statistical parameters including central tendency (e.g. means) or other basic estimates (e.g. regression coefficient) AND variation (e.g. standard deviation) or associated estimates of uncertainty (e.g. confidence intervals)
☐	☒	For null hypothesis testing, the test statistic (e.g. F, t, r) with confidence intervals, effect sizes, degrees of freedom and P value noted<br>*Give P values as exact values whenever suitable.*
☒	☐	For Bayesian analysis, information on the choice of priors and Markov chain Monte Carlo settings
☒	☐	For hierarchical and complex designs, identification of the appropriate level for tests and full reporting of outcomes
☒	☐	Estimates of effect sizes (e.g. Cohen's d, Pearson's r), indicating how they were calculated

Our web collection on statistics for biologists contains articles on many of the points above.

Software and code

Policy information about availability of computer code

Data collection	No software was used
Data analysis	kraken2 v. 2.0.9, BBduk v.38.86, seqtk v.1.3-3115-dirty, diamond v.0.9.24, bowtie2, R packages: phyloseq v. 1.10.0, ggplot2 v. 3.3.0, pheatmap v. 1.0.12, vegan v.2.5.6

For manuscripts utilizing custom algorithms or software that are central to the research but not yet described in published literature, software must be made available to editors and reviewers. We strongly encourage code deposition in a community repository (e.g. GitHub). See the Nature Portfolio guidelines for submitting code & software for further information.

Data

Policy information about availability of data

All manuscripts must include a data availability statement. This statement should provide the following information, where applicable:

- Accession codes, unique identifiers, or web links for publicly available datasets
- A description of any restrictions on data availability
- For clinical datasets or third party data, please ensure that the statement adheres to our policy

The datasets supporting the conclusions of this article are publicly available at the European Nucleotide Archive. The custom-made database is available upon request. All accession numbers are specified in Supplementary Table 4 and Supplementary Data 1. The source data behind the figures can be found in Supplementary Data 2.

Human research participants

Policy information about studies involving human research participants and Sex and Gender in Research.

Reporting on sex and gender	This information has not been collected.
Population characteristics	*Describe the covariate-relevant population characteristics of the human research participants (e.g. age, genotypic information, past and current diagnosis and treatment categories). If you filled out the behavioural & social sciences study design questions and have nothing to add here, write "See above."*
Recruitment	*Describe how participants were recruited. Outline any potential self-selection bias or other biases that may be present and how these are likely to impact results.*
Ethics oversight	*Identify the organization(s) that approved the study protocol.*

Note that full information on the approval of the study protocol must also be provided in the manuscript.

Field-specific reporting

Please select the one below that is the best fit for your research. If you are not sure, read the appropriate sections before making your selection.

☐ Life sciences ☐ Behavioural & social sciences ☒ Ecological, evolutionary & environmental sciences

For a reference copy of the document with all sections, see nature.com/documents/nr-reporting-summary-flat.pdf

Ecological, evolutionary & environmental sciences study design

All studies must disclose on these points even when the disclosure is negative.

Study description	We search for the presence and abundance of certain species and mobilizing genetic elements in fragmented metagenomic data from a large variety of environments gathered in several studies. We then compare the relative abundance and presence/not presence of the detected fragments. The aim was to find environments in which mobliization of a set of known antibiotic resistance genes have occured.
Research sample	The species chosen to be investigated was based on a publication by Ebmeyer et al. who listed the known origin species for many known antibiotic resistance genes. The data analysed was public metagenomic DNA sequence from the environments/source human, water/sediment, wastewater treatment plants, soil and animals. A total of 2496 samples were used in the study.
Sampling strategy	The samples chosen to be included in the study had to each have at least 10 million fragments each, the read length had to be at leats 75bp and the samples had to be generated by pcr free illumina shotgun sequencing.
Data collection	The data was downloaded by Fanny Berglund from the European Nucleotide Archive and NCBI.
Timing and spatial scale	Genomes for database creation was downloaded in December 2020, additional genomes used for validation was downloaded in March 2021. Metagenomic data was downloaded during spring 2021.
Data exclusions	Origin species listed in the publication by Ebmeyer et al were excluded if no complete genome was sequenced at the time of the database creation. Metagenomic data was excluded if they did not fulfill the critea listed in "Sampling strategy".
Reproducibility	All attempts to repeat the experments were successful.
Randomization	Samples were allocated into groups based on the collected metadata such as source (e.g. water, soil, human), antibiotic intake, presence of crAssphage
Blinding	The data collection was based on what was available and we wanted to use datasets from a large variety of environments. We needed the metadata information to allocate the samples into groups.

Did the study involve field work? ☐ Yes ☒ No

Reporting for specific materials, systems and methods

We require information from authors about some types of materials, experimental systems and methods used in many studies. Here, indicate whether each material, system or method listed is relevant to your study. If you are not sure if a list item applies to your research, read the appropriate section before selecting a response.

Materials & experimental systems

n/a	Involved in the study
☒	☐ Antibodies
☒	☐ Eukaryotic cell lines
☒	☐ Palaeontology and archaeology
☒	☐ Animals and other organisms
☒	☐ Clinical data
☒	☐ Dual use research of concern

Methods

n/a	Involved in the study
☒	☐ ChIP-seq
☒	☐ Flow cytometry
☒	☐ MRI-based neuroimaging
